# Massive and parallel 10 Tbit/s physical random bit generation with chaotic microcomb

**DOI:** 10.1007/s12200-023-00081-4

**Published:** 2023-09-22

**Authors:** Yuqi Hu, Qingsong Bai, Xi Tang, Wei Xiong, Yilu Wu, Xin Zhang, Yanlan Xiao, Runchang Du, Leiji Liu, Guangqiong Xia, Zhengmao Wu, Junbo Yang, Heng Zhou, Jiagui Wu

**Affiliations:** 1 College of Artificial Intelligence, Southwest University, Chongqing, 400715 China; 2 Chengdu Spaceon Electronics Corporation Ltd., Chengdu, 610037 China; 3 School of Physical Science and Technology, Southwest University, Chongqing, 400715 China; 4 Key Lab of Optical Fiber Sensing and Communication Networks, University of Electronic Science and Technology of China, Chengdu, 611731 China; 5 Center of Material Science, National University of Defense Technology, Changsha, 410073 China

**Keywords:** Physical random bit, Chaos, Microcomb

## Abstract

Ultrafast physical random bit (PRB) generators and integrated schemes have proven to be valuable in a broad range of scientific and technological applications. In this study, we experimentally demonstrated a PRB scheme with a chaotic microcomb using a chip-scale integrated resonator. A microcomb contained hundreds of chaotic channels, and each comb tooth functioned as an entropy source for the PRB. First, a 12 Gbits/s PRB signal was obtained for each tooth channel with proper post-processing and passed the NIST Special Publication 800-22 statistical tests. The chaotic microcomb covered a wavelength range from 1430 to 1675 nm with a free spectral range (FSR) of 100 GHz. Consequently, the combined random bit sequence could achieve an ultra-high rate of about 4 Tbits/s (12 Gbits/s × 294 = 3.528 Tbits/s), with 294 teeth in the experimental microcomb. Additionally, denser microcombs were experimentally realized using an integrated resonator with 33.6 GHz FSR. A total of 805 chaotic comb teeth were observed and covered the wavelength range from 1430 to 1670 nm. In each tooth channel, 12 Gbits/s random sequences was generated, which passed the NIST test. Consequently, the total rate of the PRB was approximately 10 Tbits/s (12 Gbits/s × 805 = 9.66 Tbits/s). These results could offer potential chip solutions of Pbits/s PRB with the features of low cost and a high degree of parallelism.

**Graphical Abstract:**

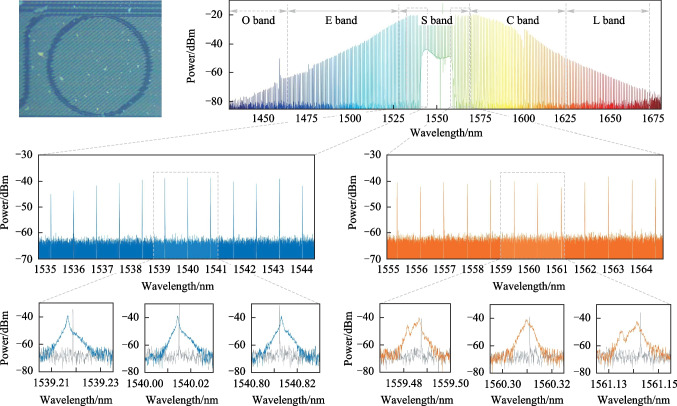

## Introduction

A faster physical random bit (PRB) is pivotal for developing large-scale tera-bit optical communication systems and Internet big-data centers. Random numbers have been applied in many fields, such as cryptography, simulation, information security, and lottery games. In recent years, the use of optical chaotic entropy sources to generate PRBs has attracted much attention owing to advantages such as large random fluctuations, high bandwidth, and easy access [1–19]. In 2008, Uchida et al. utilized a 1-bit analog-to-digital converter (ADC) and a logical XOR operation to extract PRB with a rate of up to 1.7 Gbits/s from two broadband chaotic laser beams [1]. In 2009, Reidler et al. obtained a chaotic laser output using an optical distributed feedback semiconductor faser system and performed an XOR operation and m least significant bit (m-LSB) extraction [2]. A high-speed PRB was obtained with a rate of up to 12.5 Gbits/s. In 2015, Sakuraba et al. reported the use of bandwidth-enhanced chaos to generate 1.2 Tbits/s PRBs with three-cascaded semiconductor lasers [4]. Based on silicon integration, in 2020, a PRB generator using the mesoscopic chaos from a silicon photonic crystal microcavity was proposed [13].

In terms of generating ultrafast PRB, the previous approaches used the broadband optical chaos of semiconductor lasers [1–12], such as an optical chaos with bandwidth of up to 50 GHz [5]. A 640-Gbit/s PRB signal was achieved using ultrafast detectors and ultrafast sampling of the oscilloscope. However, these methods are very expensive. Another approach is the parallel method, where the bitrate in each channel is relatively low [7–12], and the comprehensive PRB rate is still very high with multiple parallel channels. The parallel scheme can avoid the use of expensive wideband detectors and the bottleneck of ultra-high speed ADC sampling rate. However, most previous parallel schemes were non-integrated and had a small number of channels [8–12], such as 2–3 channels. Integrative and large-scale parallelism remains an open issue.

Fortunately, advanced nanophotonic technology has enabled integrated microresonators with ultrahigh *Q* factors and chip-scale microcombs [20–26]. Among various comb dynamic states, a chaotic comb has high nonlinearity. In a chaotic comb, each comb tooth exhibits a chaotic dynamic oscillation, which is suitable for PRB generation. In 2023, Chen et al. proposed a new type of lidar for the first time using a chaotic microcomb to overcome time and frequency congestion barriers [27].

There are various approaches to generation of combs, such as chaotic fiber lasers [32]. Here, we used a Si_3_N_4_ microresonator with compactness and good scalability to generate chip-scale microcombs [34]. It could simultaneously output hundreds of chaotic comb teeth through a wavelength division multiplexer and use them as entropy sources to generate massive random numbers. The randomness was verified with NIST statistical testing [28].

## Chaotic combs based on microresonator

**Fig. 1:**
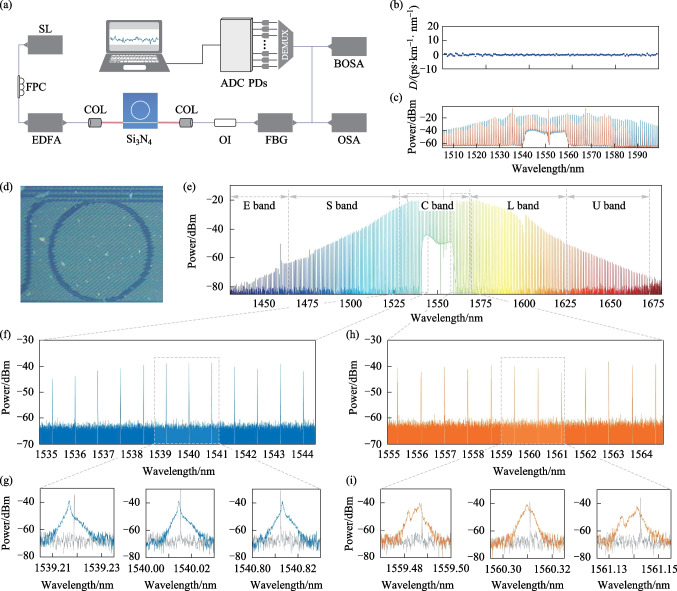
Generation of chaotic microcomb. **a** Experimental setup: *SL* semiconductor laser, *FPC* fiber polarization controller, *EDFA* erbium-doped optical fiber amplifier, *COL* collimating lens, *OI* isolator, *FBG* Fiber Bragg Grating, *PDs* photodetectors, *ADC* analog-to-digital converter, *OSA* optical spectral analyzer, *BOSA* Brillouin optical spectral analyzer. **b** Measured integrated dispersion *D*. **c** Comb evolution trace**.** Non-chaotic state (orange) and chaotic state (blue) optical spectra. **d** Image of an Si_3_N_4_ microresonator. **e** Optical spectra of chaotic microcomb generated by the Si_3_N_4_ microresonator, with a free spectral range of 100 GHz, covering 1450–1650 nm. **f** Zoom-in spectrum of the chaotic microcomb in the range of 1535–1545 nm. **g** Magnified spectrum of the chaotic microcomb tooth near 1539.21, 1540.01, and 1541.81 nm, respectively. Both chaos state (blue) and non-chaos state (gray) are presented. **h** Zoom-in spectrum of chaotic microcomb in the range of 1555–1565 nm. **i** Magnified spectrum of the chaotic microcomb tooth near 1559.48, 1560.31, and 1561.14 nm, respectively. Both chaos state (orange) and non-chaos state (gray) are presented

In the experiment, the microresonator was a specially designed Si_3_N_4_ microresonator with near-zero dispersion, which was conducive to generating broadband microcombs. The integrated dispersion of the microresonator is shown in Fig. 1b. The advantage of near-zero-dispersion microresonators is that the small group velocity dispersion (GVD) enables a small spectral deviation [33, 35]. Microresonators with a samllfree spectral range were preferred to ensure that there were sufficient comb teeth. Before generating microcombs, transmission scans of each microresonator integrated on a S_3_N_4_ chip were performed using low-power scanning laser to pump the microcavity in the wavelength range of 1500 to 1620 nm. Finally, we chose a microresonator with a size of 400 μm × 400 μm, a *Q* factor of 2.2 × 10^6^ and a free spectral range of 100 GHz, as shown in Fig. 1d. The experimental setup was shown in Fig. 1d. Because the resonator could generate a wider microcomb when the pump light energy was higher, the tunable semiconductor laser used to scan around the resonant frequency of the microresonator acts as a pump laser source, whose output power was amplified to 32 dBm with a erbium-doped fiber amplifier (EDFA). Initially, the frequency of the CW pump was in the far blue-detuned regime and was further tuned toward resonance. In Fig. 1c the orange waveform represents the non-chaotic comb, whereas the blue waveform represents the chaotic comb. During the process of tuning towards resonance, the microcomb was gradually broadened and became top-flat [39]. In the experiment, we continuously pumped the microresonator to generate chaotic microcombs, and used a temperature controller to control the temperature of the chip carrier at 37.5 °C to prevent the chip displacement under high power pumping. Temperature control of the microresonator was necessary to prevent thermal effects from changing the laser cavity detuning under a high-energy pump laser. Under stable temperature conditions, the microcombs were wider, flatter, and more stable. As seen in Fig. 1e, a chaotic microcomb was generated with a repetition frequency of 100 GHz and covering 1430–1675 nm, which spanned from the O band to the L band. The soliton state was determined to be unnecessary for the physical random bits. Compared to the soliton state [36], the chaotic state exhibited highly random variation, and was thermally self-locked [37, 38]. There was no need to enter the red-detuned regime while ensuring thermal equilibrium in the Si_3_N_4_ resonator [37]. To visually reflect the chaotic state of the microcomb, we used an ultrahigh-resolution Brillouin optical spectral analyzer (BOSA). As shown in Fig. 1f–i, the comb teeth were observed approximately 10 nm from the pump light, and three of them were selected for detailed observation. Figure 1f shows a zoom-in view of the microcomb covering wavelengths in the range from 1535 to 1545 nm. Figure 1g shows a zoom-in view of the comb teeth with wavelengths of 1539.22, 1540.01, and 1540.81 nm, respectively. In Fig. 1g, the gray waveforms represent the spectrum of the nonchaotic output at the corresponding wavelengths, whereas the color waveforms represent the detailed spectra of the comb teeth with a wider bandwidth compared to the nonchaotic case and exhibiting a chaotic state, indicating the feasibility of the proposed scheme of a random bit generator based on a microcomb. Analogously, Fig. 1h shows the zoom-in view of the microcomb in the range from 1555 to 1565 nm with free spectral range of less than 0.8 nm. Figure 1i shows a magnified view of the comb teeth at wavelengths of 1559.48, 1560.31, and 1561.13 nm, respectively. Similarly, they exhibit a chaotic state was generated.

**Fig. 2:**
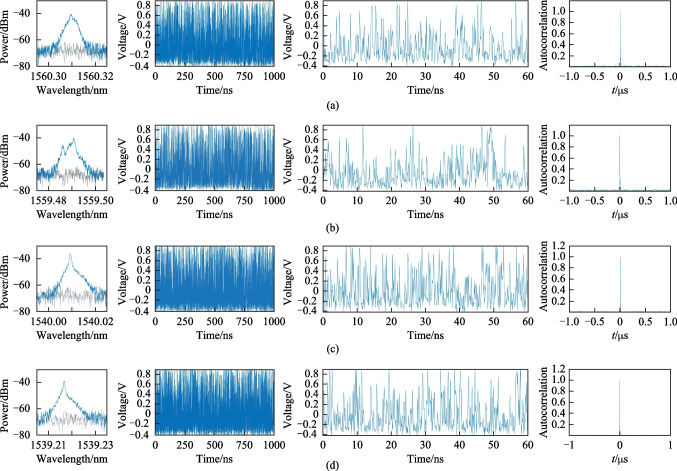
In the columns from left to right, optical spectra, the corresponding time sequence, magnified time sequence, and autocorrelation of the time sequence are presented for the of chaotic microcomb tooth at 1560.31 nm (**a**), 1559.48 nm (**b**), 1540.01 nm (**c**), and 1539.21 nm (**d**), respectively

The ultrawideband microcomb was input into a demultiplexer (DEMUX), and each comb tooth was filtered and collected by the corresponding avalanche photo diodes (APDs). The analog signal was converted into a digital signal through analog-to-digital converters (ADCs) with a sampling rate of 10 G samples per second (SPS) and a bandwidth of 2.5 GHz, then transmitted to the computer for post-processing. As shown in Fig. 2a, the comb tooth at 1560.31 nm was filtered out by a filter and monitored by the BOSA and APD. The first subplot of Fig. 2a shows the optical spectrum of the chaotic tooth at 1560.31 nm. The second and third subplots in Fig. 2a show the temporal signal of the chaotic comb tooth and a zoom-in view of the temporal signal. The temporal signal exhibited a chaotic state. The time delay (TD) signature of chaotic signals affects the generation of random bit sequence [1, 2]. Therefore, in this experiment, autocorrelation analysis was used to evaluate the TD signature of the chaotic comb signals, as shown in the fourth subplot of Fig. 2a. The time corresponding to the autocorrelation peak was 0.01 μs, indicating the TD signature was not obvious. This was conducive to the generation of random bit sequences. As shown in Fig. 2b–d, the chaotic teeth with wavelengths of 1559.48, 1540.01, and 1539.21 nm were filtered out simultaneously. The same analysis was performed on these teeth. The results showed that all the comb teeth exhibited a chaotic state in the optical and temporal domains, and the TD signatures were not obvious.

## Random bit generation

**Fig. 3:**
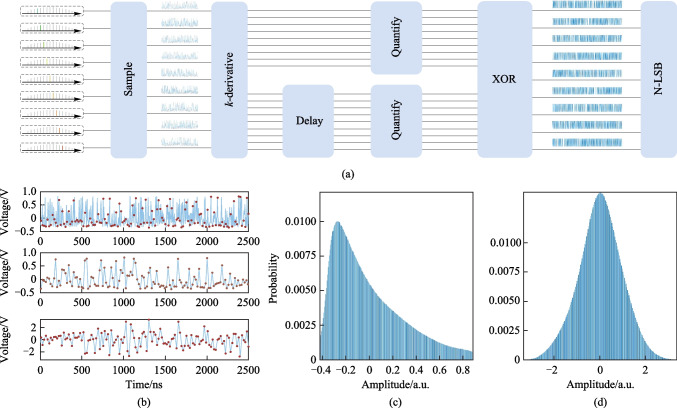
**a** Flow chart of post-processing for the entropy source of chaotic comb tooth. **b** From top to bottom, the sub-figures show the results of original data, the 4-point sampled data and the data after discrete derivative operation respectively. The blue lines show the time series and the red dots correspond to the sampling points. **c** and **d** show the Histogram of data before and after discrete derivative operation respectively

A flowchart of the post-processing method for the entropy source of the chaotic comb tooth is shown in Fig. 3a. First, owing to the limitation of chaotic bandwidth, the original chaotic data acquired by the ADC needed to be sampled at a 4-point sampling interval and then subjected to* k*th-order discrete derivative; in the experiment, we performed a fourth-order discrete derivative on the sampled data sampled, and the selected unit buffer time was 79.2 ns. The specific equation is as follows:

1 $$D(t) = \sum\limits_{i = 0}^{2k - 2} {\left\{ {{{( - 1)}^i}C_{2k - 2}^id(\Delta \times i + t)} \right\}} ,$$

where *d*(*t*) and *D*(*t*) represent the sampled chaotic data sequence and the data sequence after *k*-order discrete derivative differentiation, respectively. Δ represents unit buffer time.* C* represents combination operation. Owing to the highly symmetric distribution of the results caused by discrete derivative operations, the problem of bias, is also eliminated [29]. Taking the *k*th discrete derivative of the original data were also beneficial for increasing the least significant bit (LSB), thereby improving the generation rate of the PRB [29]. Each discrete derivative operation increased the original data by an additional bit. After undergoing discrete derivative, the processed data were subjected to a self-delay operation, and then the non-delayed and delayed signals were quantified with 8 + *k* bits, where *k* is the order of the derivative operation mentioned above. The non-delayed quantization data and delayed quantization data underwent binary conversion to generate the non-delayed data bit sequence and delayed data bit sequence. Finally, we intercepted the *N*-bit LSB of these two bit sequences and performed bitwise XOR operations [30]. Figure 3b shows the results obtained in the post-processing process described above. The upper panel indicates the original data sampled by the ADC, and the middle panel shows the results of sampling the original chaotic data at a 4-point sampling interval. The lower panel indicates the results after the *k*th-order discrete derivative. Figure 3c shows the probability distribution of the original chaotic data. After *k*th-order discrete derivative operation, the distribution of the data exhibited a standard Gaussian distribution, as shown in Fig. 3d.

**Fig. 4:**
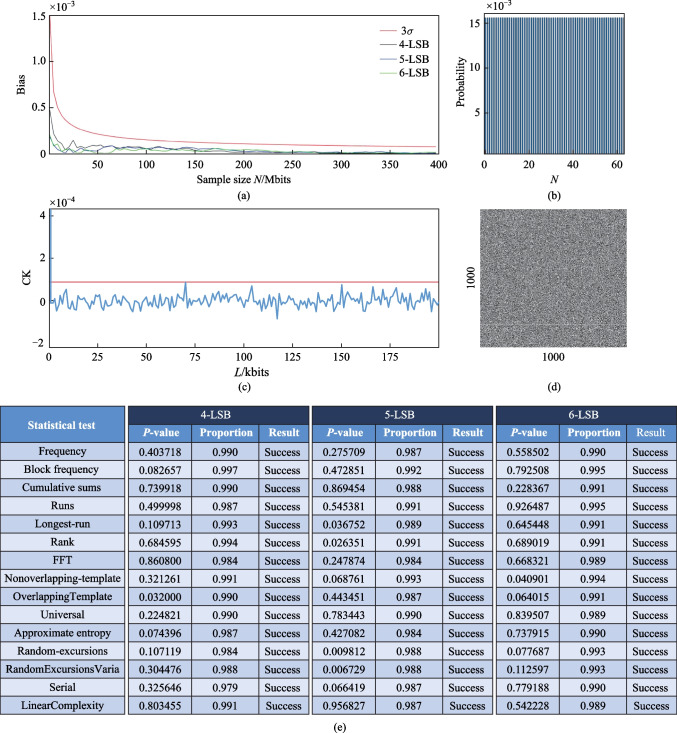
Analysis results of the bit sequence obtained from an entropy source at 1560 nm. **a** Variation of statistical bias with sample number *N* for the 4-LSB, 5-LSB, and 6-LSB case, and the standard 3*σ* curve; **b** histogram of final data sequence after 6-LSB post-processing; **c** autocorrelation coefficient (CK, blue) of first 200 kbits of the bit sequence generated under 6-LSB and the standard 3*σ* curve (red); **d** 2D black and white image generated by the first 1 M points in the bit sequence under 6-LSB processing in the form of 1000 × 1000, where bit “1” and bit “0” are converted into white and black dots, respectively. **e** Results of the NIST tests for bit sequences after 4-LSB, 5-LSB, and 6-LSB post-processing, where a set of 1000 sequences with 1 M bit is used for evaluation

In Fig. 4a, the statistical deviation of the random sequence for the 4-, 5-, and 6-LSB cases are shown, as analyzed after the XOR operation, where the length of the LSB directly influenced the rate of the random bit generator. The red curve in Fig. 4a represents the standard curve of the statistical deviation, and it is calculated as follows:

2 $${3}\sigma = \frac{1.5}{{\sqrt N }},$$

where *N* is the number of detected sampling points. The other curves represent the statistical deviation curves for generating bit sequences in different LSB cases. Their calculation formula can be expressed as *B* = |*P*_1_ − 0.5|, where *P*_1_ is the probability of 1 appearing in the bit sequence [31]. When the statistical deviation curve of a bit sequence crosses the standard curve, the bit sequence is not qualified. Figure 4b shows a histogram of the final data sequence after 6-LSB post-processing. In the histogram, the distribution of decimal numbers converted from binary numbers after 6-LSB post-processing is almost unbiased. We also performed an autocorrelation analysis of the bit sequence, by using the equation as follows:

3 $${\text{autocor}}({\Delta }) = \frac{{\overline {S(t + {\Delta }) - S(t)} * (S(t) - \overline {S(t)} )}}{{\sqrt {{{(\overline {S(t + {\Delta }) - S(t)} )}^2} * \overline {{{(S(t) - \overline {S(t)} )}^2}} } }},$$

where *S*(*t*) represents bit sequence, and Δ represents time shift. The $\overline {S(t)}$ represents average value of *S*(*t*). In the Fig. 4c, The results show that the autocorrelation coefficient of the bit sequence is lower than the standard 3*σ* curve. Figure 4d shows a 2D black and white image generated by the first 1 M points in the bit sequence under 6-LSB processing in the form of 1000 × 1000. Bits “1” and “0” are represented by white and black dots, respectively. It is evident that the distribution of black and white pigments in this two-dimensional image was uniform, and there was significant randomness. NIST testing was used for the bit sequences that passed statistical bias test and autocorrelation test to evaluate the statistical randomness of the bit sequences. For the “success” of the passing NIST test, the *p*-value should be greater than 0.0001, and the proportion should be within the range of 0.99 ± 0.0094392, using 1000 samples of 1 M bit data [28]. For tests that generated multiple* p*-values and proportions, the worst-case scenario was provided. As shown in Fig. 4e, the experimental bit sequences of 4-LSB, 5-LSB, and 6-LSB passed all 15 NIST tests, indicating that the random bits were qualified. Under the conditions of 4-point sampling, 4th order discrete derivative, and 6-LSB, the generated bit sequence had a rate of 12 G (6 bits × 2 GHz).

**Fig. 5:**
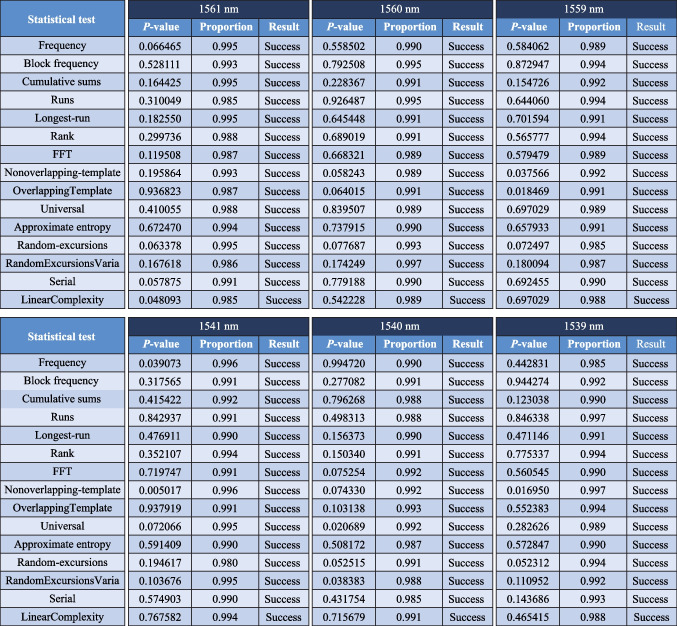
Results of the NIST Tests for bit sequences generated by the entropy source of chaotic comb tooth at 1539, 1540, 1541, 1561, 1560, and 1559 nm, respectively, after 6-LSB post-processing. A set of 1000 sequences with 1 M bits is used for evaluation

As shown in Fig. 5, to avoid specificity of the data from a single comb tooth, it was necessary to analyze multiple comb teeth in the experiment. We selected six comb teeth at 1561, 1560, 1559, 1539, 1539, and 1540 nm, respectively, on the left and right sides of the pump light wavelength and processed these comb teeth using the same data post-processing method depicted above to generate a random bit sequence. NIST testing was then conducted, and the results showed that all six comb teeth passed the 15 NIST tests with 6-LSB. Therefore, using 6-LSB as a condition for qualified PRB and assuming that all comb teeth can pass through the NIST test, considering that with 294 teeth of the microcomb obtained in the experiment ranging from 1430 to 1675 nm, the rate of PRB could reach 3.528 Tbit/s (12 Gbits/s × 294 = 3.528 Tbits/s).

**Fig. 6:**
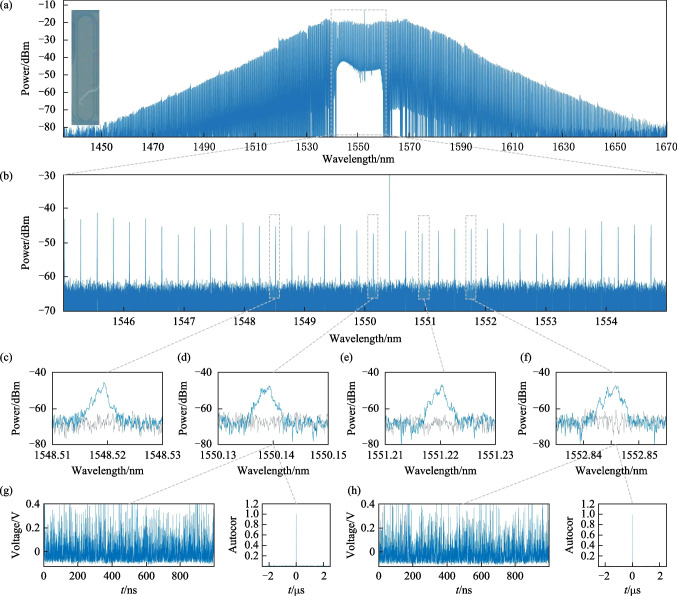
Generation of chaotic microcomb by using a microresonator with *Q* factor of 2.8 × 10^6^. **a** Optical spectra of chaotic microcomb generated by microresonator with free spectral range of 33.6 GHz covering the wavelength range of 1450–1670 nm. The inset shows an micrograph of the microresonator. **b** Close-up spectrum of the chaotic microcomb in the wavelength range of 1545–1555 nm. **c** Zoom-in spectrum of the chaotic microcomb in the range of 1548.1–1548.53 nm. **d** Zoom-in spectrum of the chaotic microcomb in the range of 1550.13–1559.15 nm. **e** Zoom-in spectrum of the chaotic microcomb in the range of 1551.21–1551.23 nm. **f** Zoom-in spectrum of the chaotic microcomb in the range of 1552.84–1552.85 nm. **g** Time sequence of the chaotic microcomb at 1550.13 nm and autocorrelation of the time sequence. **h** Time sequence of chaotic microcomb at 1552.84 nm and autocorrelation of the time sequence

Figure 6a shows the spectrum of a wider and denser microcomb generated by another microresonator, whose micrograph is shown in the inset. Compared to the previous microresonator, this microresonator could generate nearly three times the number of comb teeth, with a *Q* factor of 2.8 × 10^6^, and a free spectral range of only 33.6 GHz. In the experiment, a 1550.799 nm pump light source with an optical power of 31 dBm was used to pump the microresonant cavity. The microcomb could range from 1430 to 1670 nm with smaller free spectral range and more chaotic comb teeth. In Fig. 6b, we used a high-precision spectrometer BOSA to observe and collect microcombs in the wavelength range of 1545–1555 nm, and it can be seen that the wavelength spacing between the comb teeth is similar, and it is approximately 0.25 nm. Meanwhile, as shown in Fig. 6c–f, we selected four comb teeth at 1548.53, 1550.14, 1551.23, and 1552.85 nm respectively for detailed observation. Using a BOSA, one can see that the original narrow laser spectrum widens from a non-chaotic state to a chaotic state, after passing through the microresonator, indicating the feasibility of generating effective random numbers.

**Fig. 7:**
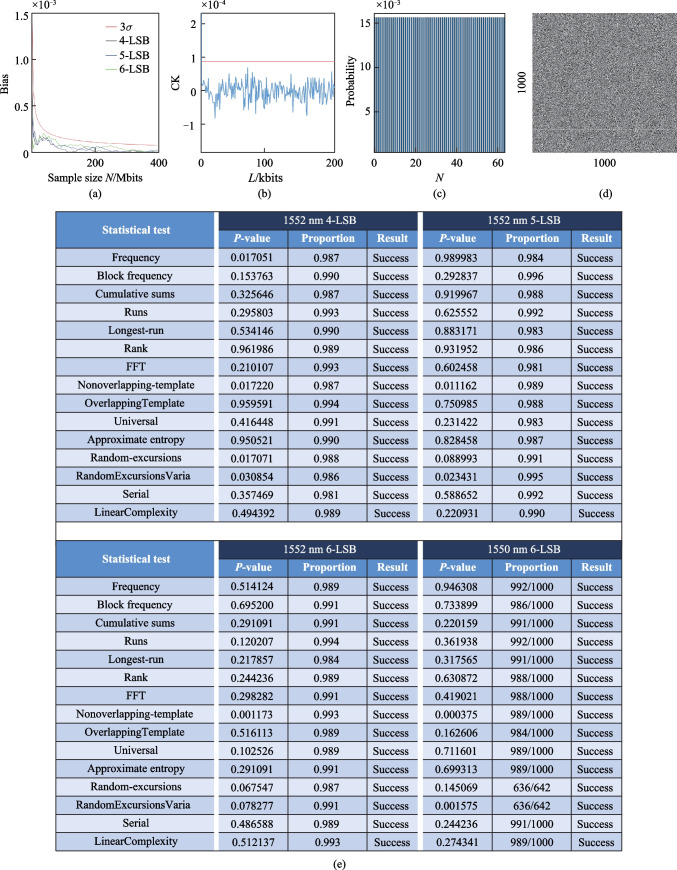
Analysis of the bit sequence generated by an entropy source at 1560 nm. **a** Variation of statistical bias with sample number *N* for 4-LSB, 5-LSB, and 6-LSB cases, and the standard 3*σ* curve. **b** Autocorrelation coefficient (CK) of the first 200 kbits of the bit sequence generated after 6-LSB post-processing and the standard 3*σ* curve. **c** Histogram of the final data sequence of after 6-LSB post-processing. **d** The 2D graph generated by the first 1 M points of the bit sequence after 6-LSB post-processing in the form of 1000 × 1000, where bit “1” and bit “0” are represented by white and black dots, respectively. **e** Results of the NIST tests for the bit sequences after 4-LSB, 5-LSB, and 6-LSB post-processing, using the chaotic comb teeth at wavelengths of 1550.13 and 1552.84 nm respectively. A set of 1000 sequences of 1 M bits is used for evaluation

A comb tooth at wavelength of 1552.84 nm was filtered out. Subsequently, its time series were collected to generate a random bit sequence using the above-mentioned post-processing scheme. As shown in Fig. 7a, the statistical bias of random sequences in different LSB cases was analyzed. Red represents the standard curve of statistical deviation, whereas the other curves represent the statistical deviation curves for generating bit sequences in different LSB cases. None of the curves for different LSB cases crossed the standard curve. Figure 7b shows the autocorrelation coefficient of the bit sequence for the 6-LSB case. The results show that the autocorrelation coefficient of the bit sequence did not exhibit delay characteristics is lower than the standard 3*σ* curve. Figure 7c shows histogram of the final data sequence after 6-LSB post-processing. In the histogram, the distribution of decimal numbers converted from binary numbers after 6-LSB post-processing is almost unbiased. Figure 7d shows a two-dimensional graph generated by the first 1 M points in the bit sequence after 6-LSB post-processing in the form of 1000 × 1000.

We also conducted NIST testing on bit sequences after 4-LSB, 5-LSB, and 6-LSB post-processing. Figure 7e shows that the bit sequence in the 4-LSB, 5-LSB, and 6-LSB cases passed all the 15 NIST statistical tests. We filtered out the comb tooth at wavelength of 1550.18 nm for further analysis. The bit sequence for the 6-LSB case passed all 15 NIST statistical test. Therefore, using 6-LSB as a condition for qualified PRB and assuming that all comb teeth can pass through the NIST test, with 805 teeth in microcomb covering the wavelength range of 1430–1670 nm, the rate of PRB could reach 9.66 Tbits/s (12 Gbits/s × 805 = 9.66 Tbits/s).

## Conclusion

In conclusion, we experimentally developed a massive PRB generator based on a chaotic microcomb using a microresonator. In the experiment, the chaotic microcomb with a free spectral range of 100 GHz, covering 1430–1675 nm, was first generated using a Si_3_N_4_ microresonator. Several chaotic comb teeth were filtered out as entropy sources to generate a PRB sequence, and the temporal signals of the chaotic comb teeth were converted into digital signals with ADCs. Next, XOR operation and *N*-LSB extraction methods were used to generate qualified and ultrafast random bit sequences, which were then tested with the NIST SP 800-22 test. The results showed that they all successfully passed the standard test. Assuming all 294 comb teeth of the chaotic comb successfully passed the standard test, the generation rate of the PRB generator reached approximately 4 Tbit/s. Moreover, another Si_3_N_4_ microresonator was used to generate a microcomb with a free spectral range of 33.6 GHz and more chaotic comb teeth, covering a range of wavelengths from 1430 to 1670 nm. With the same processing method, the PRB sequence generated from the microcomb also successfully passed the NIST standard test, resulting about 10 Tbit/s generation rate. Our experimental results offer a possible integration solution for ultra-fast PRB generation with high degree of parallelism and low cost. It can be useful in next generation ultra-speed communication systems and big-data processing centers.

## Data Availability

Data underlying the results presented in this paper are not publicly available at this time but may be obtained from the authors upon reasonable request.
